# Microplate Chemiluminescent Assay for DNA Detection Using Apoperoxidase-Oligonucleotide as Capture Conjugate and HRP-Streptavidin Signaling System

**DOI:** 10.3390/s18041289

**Published:** 2018-04-23

**Authors:** Ivan Sakharov

**Affiliations:** Department of Chemistry, Lomonosov Moscow State University, 119991 Moscow, Russia; sakharovivan@gmail.com; Tel.: +7-495-939-3407

**Keywords:** DNA, enzyme-linked assay, capture conjugate, peroxidase, chemiluminescence

## Abstract

A covalent conjugate of horseradish apoperoxidase and amino-containing oligonucleotide was synthesized for the first time. Using the obtained conjugate as a capture reagent chemiluminescent microtiter plate-based assay for detection of 35-mer fragment of hepatitis B virus (HBV) DNA (proof-of-concept analyte) was developed. To detect the target DNA, a signaling system consisted of biotinylated reporter oligonucleotide and HRP-streptavidin conjugate was used. The high sensitivity of the assay was due to the enhanced chemiluminescence reaction, where 3-(10′-phenothiazinyl)propane-1-sulfonate/*N*-morpholinopyridine pair was used as an enhancer. Under the optimized conditions the limit of detection and a working range of the assay were 3 pM and 6–100 pM, respectively. The assay sensitivity was 1.6 × 10^5^ RLU/pM of target. The coefficient of variation (CV) for determination of HBV DNA within the working range was lower than 6%.

## 1. Introduction

The selective detection of DNA is of great significance for the early diagnosis of disease, mutation and pathogen detection, food identification and forensic analysis. Since in real samples the DNA concentration is low, amplification of the target molecule is usually applied prior to its determination. Polymerase chain reaction (PCR) is a technique used in molecular biology to amplify a single copy or a few copies of DNA segment across several orders of magnitude generating thousands to millions copies of a particular DNA sequence [1,2]. Besides PCR, some isothermal amplification methods are also used in nucleic acid analysis [1,3,4,5]. 

The methods of DNA detection are commonly based on a hybridization reaction [6,7,8,9]. These methods can be divided on homogeneous and heterogeneous ones. Although the homogeneous assays are rapid and simple [10,11], heterogeneous methods have higher sensitivity. Moreover, heterogeneous assays are less affected by the matrix effect. Therefore, the heterogeneous methods of DNA detection attract much attention by analytical chemists. 

Such assays are usually constructed on the basis of a sandwich format, where the capture DNA is immobilized on the solid surface, which then subsequently reacts with the target DNA and the labeled reporter DNA. In electrochemical assays, the capture DNA is adsorbed on the electrode surface [12,13]. In case of gold electrodes, the capture DNA is chemically bound using Au-S bonds [14,15]. The optical methods of DNA detection are usually developed with the use of gold or carbon nanoparticles (NPs) [16,17,18,19] and magnetic beads [20,21]. An alternative solid carrier for DNA detection are microtiter plates [9,22,23], which are widely applied in the production of ELISA kits. 

Comparison of solid carrier properties showed that NPs binding surface is higher than that of microtiter plates wells. However, at the use of highly active signaling systems, for example, the enhanced chemiluminescence reaction catalyzed plant peroxidases [24,25], limited surface of the microplates is not an obstacle for the development of sensitive DNA assays [26]. Also, it should be noted that complicated bioconjugation and separation of NPs, their relatively low yield, poor re-dispersion, high cost and potential environmentally unfriendly impact limit their application [27]. In contrast, microtiter plates are free from the mentioned shortcomings. As demonstrated previously, commercially available microtiter plates are a highly standardized product which allows a determination of different analytes with high accuracy and precision. Moreover, microplate-based assays can be easy automatized. Despite the above properties of microtiter plates, presently they are, undeservedly, rarely used in nucleic acid assays. 

Some methods of DNA immobilization on the surface of microtiter plates have been reported. One of them is the direct adsorption of unmodified oligonucleotide DNA on microplate surface [28,29]. However, in this case the weak signal-to-noise ratio and the very low reproducibility were observed [30]. Moreover, there is a possibility of leakage of capture oligonucleotide as a result of its interaction with target, if the same fragment of the oligonucleotide is responsible both for the immobilization on support and the formation of complex with target.

Covalent binding of DNA oligonucleotides on the microplate surface is also used. Presently the microtiter plates with different activated groups are commercially available and can be used for immobilization of amino-containing oligonucleotides [9,31]. The main drawback of such plates is their high cost. 

Another method is based on the interaction of its biotinylated derivative of DNA with streptavidin preliminary adsorbed on the plate surface. This approach successfully allowed the development of some sensitive DNA assays [26,32,33,34].

Presently conjugates of streptavidin with horseradish peroxidase (HRP) and polyHRP are commercially available. By this, it is advantageous to use the biotin-streptavidin pair not for DNA immobilization, but in the signaling system. Since it is impossible to use the biotin-streptavidin pair both in capture and in signaling systems simultaneously, in order to use steptavidin conjugates it is necessary to develop alternative methods of DNA immobilization. 

Here, we describe an alternative approach to the immobilization of capture DNA oligonucleotide on the microtiter plate. Since polystyrene microtiter plates have a high binding capacity to adsorb proteins, we used a conjugate of horseradish apoperoxidase and DNA (apoHRP-CAP) as a capture conjugate. This conjugate was synthesized upon the reaction of aldehyde groups of the oxidized glycoprotein with amino-containing oligonucleotide by Nakane’s method. The obtained conjugate was successfully used in the microplate-based sandwich method for determination of 35-mer fragment of hepatitis B virus (HBV) DNA used as a proof-of-concept analyte. A non-covalent complex of biotinylated reporter DNA (b-REP) and streptavidin-HRP conjugate was used as the signaling system. To improve assay sensitivity, HRP activity was measured towards luminol/H_2_O_2_ substrates using 3-(10′-phenothiazinyl)propane-1-sulfonate/*N*-morpholinopyridine pair as an enhancer of peroxidase-catalyzed chemiluminescence [35,36].

## 2. Materials and Methods

### 2.1. Chemicals

Horseradish peroxidase (HRP, isoenzyme c, RZ 3.0), luminol, Tween-20, Tris-HCl, casein, sodium periodate and NaBH_4_ were purchased from Sigma (St. Louis, MO, USA). Sodium 3-(10′-phenothiazinyl)propane-1-sulfonate was synthesized as previously described [37]. 4-Morpholinopyridine was from Aldrich (St. Louis, MO, USA); H_2_O_2_ (30%) was from ChimMed (Moscow, Russia). Streptavidin-HRP conjugate was a gift of Dr. E.E. Efremov (Russian Cardiology Research Center, Moscow, Russia). All salts were of analytical or chemical purity grade.

All DNA oligonucleotides (35-mer fragment of HBV DNA, 5′-TGG GAG GAG TTG GGG GAG GAG ATT AGG TTA AAG GT-3′; amino-containing capture DNA, 5′-(NH_2_) TT TTT ACC TTT AAC CTA ATC TCC TC-3′; biotinylated reporter DNA, 5′-CCC CAA CTC CTC CCA TTA GAA G (biotin)-3′; amino-containing reporter DNA, 5′-CCC CAA CTC CTC CCA TTA GAA G (NH_2_)-3′ were purchased from Sintol (Moscow, Russia).

The concentration of HRP was measured by using ε_402_ = 102 000 M^−1^ cm^−1^ and ε_280_ = 34,000 M^−1^ cm^−1^ [38]. The H_2_O_2_ concentration was determined by monitoring A_240_ using ε = 43.6 M^−1^ cm^−1^ [39]. The required dilutions of H_2_O_2_ were prepared daily.

### 2.2. Preparation of Horseradish Apoperoxidase

Horseradish apoperoxidase was prepared by the extraction of heme from native HRP as previously described [40]. The absence of absorbance at Soret band in the UV-vis spectrum of the obtained apoHRP confirmed the completeness of heme extraction from HRP (Figure S1). Moreover, apo-HRP had no enzyme activity measured towards 2,2′-azinobis-(3-ethylbenzthiazoline-6-sulfonic acid) [41].

### 2.3. Synthesis of Conjugates of apoHRP and Capture Oligonucleotide

Capture oligonucleotide was conjugated with apoHRP as follows: apoHRP (4 mg/mL) was oxidized with 17 mM sodium periodate for 20 min in the dark at 25 °C [42]. After the dialysis against 1 mM acetic buffer, pH 4.5 (overnight at 4 °C), 240 µL of the oxidized apoHRP was mixed with 30 μL of the amino-containing capture oligonucleotide and 30 μL of 1 M carbonate buffer, pH 9.6, and stirred for 3 h at room temperature. The concentration of apoHRP in the reaction mixture (ε_280_ = 34,000 M^−1^ cm^−1^) was 1 × 10^−5^ M; CAP concentrations were 2 × 10^−6^ M, 1 × 10^−5^ M and 3 × 10^−5^ M. To reduce the azomethine bonds, 10 μL NaBH_4_ solution (4 mg/mL) was added to the reaction mixture. The obtained solutions were incubated for 1 h in the dark at 25 °C and then were intensively dialyzed against 10 mM Tris-HCl, pH 8.0. To separate the obtained conjugates and excess of the capture DNA, ultrafiltration on Amicon Ultra 0.5 (30 K) was applied. The purified conjugates were stored at −20 °C or at 4 °C after their freeze-drying. 

### 2.4. Synthesis of Conjugates of HRP and Reporter Oligonucleotide

HRP-REP conjugates were synthesized by the procedure described above for the synthesis of apoHRP-CAP conjugates using apoHRP and amino-containing capture DNA instead of HRP and amino-containing reporter DNA as reagents.

### 2.5. Determination of HBV DNA by Microtiter Plate Chemiluminescent Assay

#### 2.5.1. Using HRP-REP Conjugate

The determination of target DNA was carried out using 96-wells black polystyrene microplates (High Binding, Corning, New York, NY, USA). The plates were coated by adding to each well 50 μL of apoHRP-CAP (1.5 μg/mL solutions were prepared by dissolving freeze-dried preparations in 50 mM carbonate buffer, pH 9.5) and incubated at 4 °C overnight. The plates were then washed three times using 10 mM Tris-HCl, pH 7.2 with 300 mM NaCl and 0.05% Tween 20 (TBST). To block the unoccupied sites of microplate wells, 100 μL of milk casein (1 mg/mL) in 10 mM Tris-HCl, pH 7.2 with 300 mM NaCl (TBS) were subsequently added to the wells and incubated for 1 h at 37 °C. The plates were then washed three times with TBST. Afterwards, 50 μL of the target DNA (0–4.0 nM in TBS), preliminarily annealed at 88 °C for 15 min and cooled to room temperature for 60 min, were added to the wells and incubated for 1 h at 37 °C. The plates were thrice washed with TBST. Then, 50 μL of HRP-REP (10 nM) in TBST were added to the wells. The plates were incubated for 1 h at 37 °C and then washed as described above. Finally, 100 μL of freshly prepared substrate solution (80 mM Tris, pH 8.3, containing 0.17 mM luminol, 2.1 mM 3-(10′-phenothiazinyl)propane-1-sulfonate, 8.75 mM N-morpholinopyridine, and 1.75 mM H_2_O_2_ [35]) were added to each well and stirred. Chemiluminescence was monitored at room temperature on the microplate luminometer SpectraMax L (Molecular Devices, San Jose, CA, USA).

#### 2.5.2. Using Biotinylated Reporter DNA and HRP-Streptavidin Conjugate

All the initial steps including the reaction with HBV DNA were identical to those described above for the assay with HRP-REP. Afterwards, 50 μL of biotinylated REP (100 pM in TBS), preliminarily annealed at 88 °C for 15 min and cooled to room temperature for 60 min, were added to the wells. The plates were incubated for 1 h at 37 °C and then washed with TBST three times. Afterwards, 50 μL of streptavidin-HRP (dilution 1:500) in TBS with 1 mg/mL casein were added to the wells. The plates were incubated for 1 h at 37 °C and then washed as described above. Finally, 100 μL of freshly prepared substrate solution were added to each well and stirred. Chemiluminescence was monitored at room temperature on the luminometer. 

## 3. Results and Discussion

### 3.1. Design Principle of DNA Sensing

**Figure 1:**
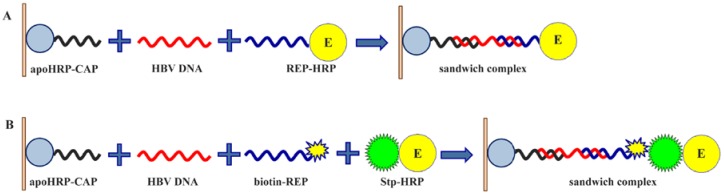
Schematic illustration of the microplate chemiluminescent assays for HBV DNA detection using (**A**) HRP-REP conjugate and (**B**) biotinylated REP and HRP-streptavidin conjugate as signaling systems.

The scheme of the microplate sandwich assay for the detection of 35-mer fragment of HBV DNA is presented in Figure 1. As a capture conjugate, apoHRP-CAP was used in this assay. Earlier covalent DNA-protein conjugates were not used as capture DNA in microplate-based assays. After adsorption of apoHRP-CAP conjugate, unoccupied surface of the microplate wells was blocked with 0.1% casein. It allowed preventing a non-specific adsorption of the used oligonucleotides and HRP-streptavidin conjugate on well surface. Then the target DNA formed a duplex with the capture oligonucleotide, because these sequences were partially complementary. To detect the bound analyte, two signaling systems were tested. In the first case (Figure 1A) HRP-REP conjugate reacted with the target DNA preliminary bound with apoHRP-CAP conjugate. In another case (Figure 1B), the detection was carried out in two steps: b-REP reacted first with the target DNA and afterwards the obtained complex reacted with HRP-streptavidin conjugate. Given the limited microplate surface, highly sensitive methods of label detection are necessary for DNA microplate assays. By this, an enhanced chemiluminescence reaction was applied to measure HRP activity. In the chemiluminescent method, luminol and hydrogen peroxide were used as HRP substrates and 3-(10′-phenothiazinyl)propane-1-sulfonate/*N*-morpholinopyridine pair—as an enhancer. It should be noted that 3-(10′-phenothiazinyl) propane-1-sulfonate/*N*-morpholinopyridine pair as well as 3-(10′-phenothiazinyl) propionic acid/*N*-morpholinopyridine pair [25,35] are the most potent enhancers of HRP-catalyzed chemiluminescence known so far, which makes this method extremely sensitive.

### 3.2. Optimization of the Synthesis of Coating Conjugate

Previously some protein-DNA conjugates have been produced using different chemical methods [43]. In our work, we successfully prepared an apoHRP-CAP conjugate using the reaction of amino-containing oligonucleotide (CAP) and apoHRP with the oxidized carbohydrate chains by Nakane’s method. This synthetic approach is widely used in the preparation of HRP-protein conjugates [44], although it was never used in the synthesis of protein-DNA conjugates. 

It should also be noted that the use of other glycoproteins, such as ovalbumin and human IgG, did not permit the production of active capture conjugates. We assume that the failure in the synthesis of active conjugates with use of ovalbumin and human IgG as protein carriers was connected with a low content of carbohydrates in these glycoproteins, which is 3.5% [45] and 2.9% [46], respectively. In turn, this fact resulted in a low concentration of aldehyde groups formed upon NaIO_4_ treatment of glycoproteins and a low yield of the reaction of interest. In contract, the sugar content in HRP was significantly higher (21.8%) [47]. Therefore, upon the synthesis of capture conjugates by the proposed method, glycoproteins with high sugar content should be used.

**Figure 2:**
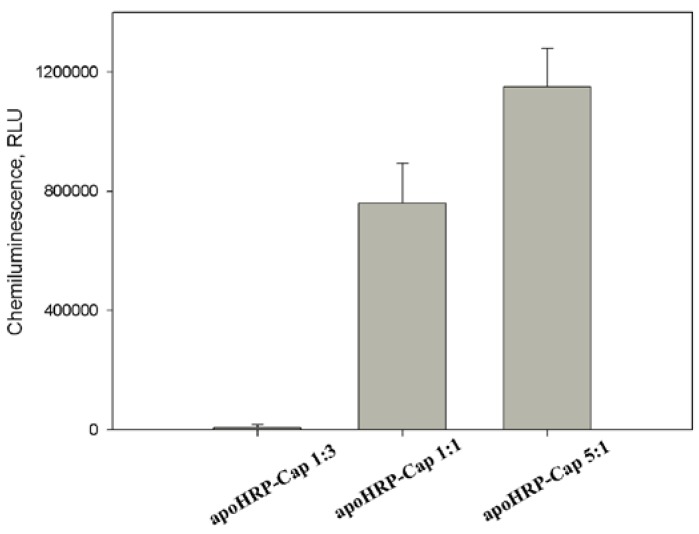
Effect of a molar ratio of the reacting compounds at the synthesis of apoHRP-CAP on the activity of the produced conjugates. Experimental conditions: concentrations of apoHRP-CAP, HBV DNA and HRP-REP were 5 μg/mL, 1 nM and 10 nM, respectively. Chemiluminescence was measured 8 min after the initiation of luminol oxidation. Each measurement was repeated thrice.

To optimize the experimental conditions of apoHRP-CAP synthesis, the concentrations of the reacting compounds in the reaction mixture were varied. The obtained results showed that the increase of a molar ratio of concentrations of apoHRP and capture oligonucleotide resulted in the increase of the efficiency of the obtained conjugates in the assay (Figure 2). The conjugate prepared at apoHRP/CAP ratio equal to 5:1 showed the highest activity. The lower activity obtained for apoHRP-CAP conjugates 1:1 and 1:3 likely connects with that oligonucleotide strands, after their conjugation with the protein, interfere each other to react with the analyte.

### 3.3. Optimization of HBV DNA Assay

In our work we compared two HRP signaling systems. The first system was based on the use of HRP-REP conjugate (Figure 1A). The synthesis of HRP-REP conjugate was described previously [26]. To optimize assay conditions, the concentration of apoHRP-CAP (5:1) varied in the coating solution. The chemiluminescent intensity strongly depended upon the concentration of apoHRP-CAP (5:1). The obtained resulted showed that the optimal concentration of this conjugate was 1.7 μg/mL.

**Figure 3:**
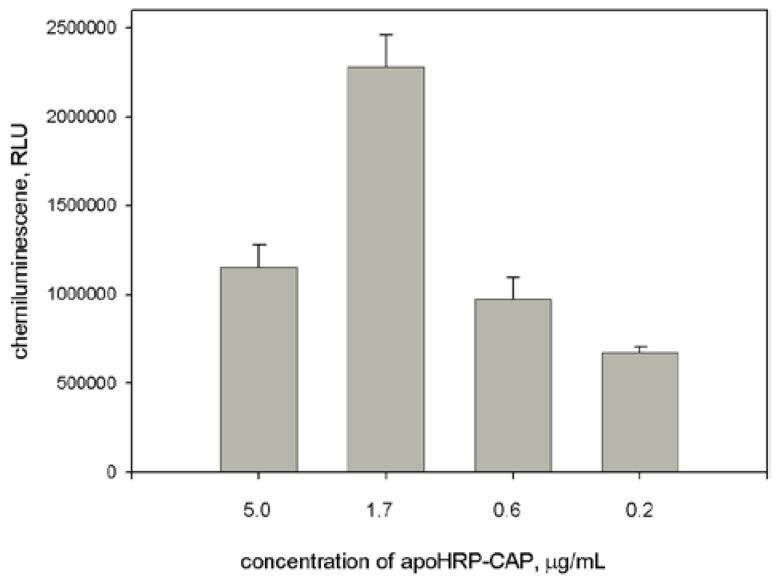
Dependence of light output upon concentration of apoHRP-CAP (5:1) at use of HRP-REP as a signaling conjugate. Experimental conditions: concentrations of HBV DNA and HRP-REP were 1 nM and 10 nM, respectively. Chemiluminescence was measured 8 min after the initiation of luminol oxidation. Each measurement was repeated thrice.

As seen in Figure 3, there is the optimal concentration of the coating conjugate and its excess leads to decrease the signal value. This likely connects with the fact that a high density of the coating molecules on microplate surface may interfere in their interaction with the analyte. Furthermore, from concentrated solutions, multilayer adsorption of the conjugate can occur. In turn, the formation of the conjugate aggregation may also prevent the interaction of the conjugate and the analyte. Earlier similar dependences have been described for ELISA assays.

**Figure 4:**
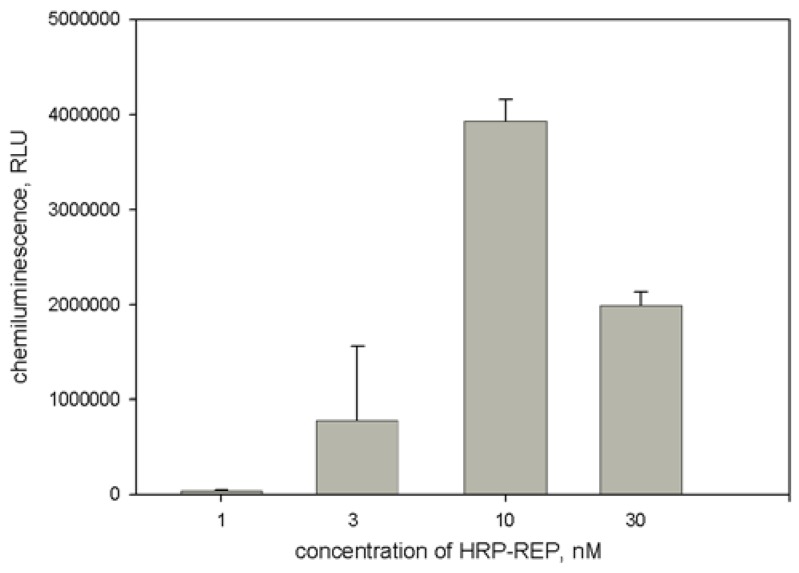
Dependence of light output upon concentration of HRP-REP conjugate. Experimental conditions: concentrations of apoHRP-CAP (5:1) and HBV DNA were 1.7 μg/mL and 1 nM, respectively. Chemiluminescence was measured 8 min after the initiation of luminol oxidation. Each measurement was repeated thrice.

At the next step the concentration of HRP-REP conjugate was optimized. As seen in Figure 4, the dependence of light output upon HRP-REP had a bell-like behavior. Since the highest intensity was obtained at HRP-REP concentration equal to 10 nM, in the further work this concentration was used.

**Figure 5:**
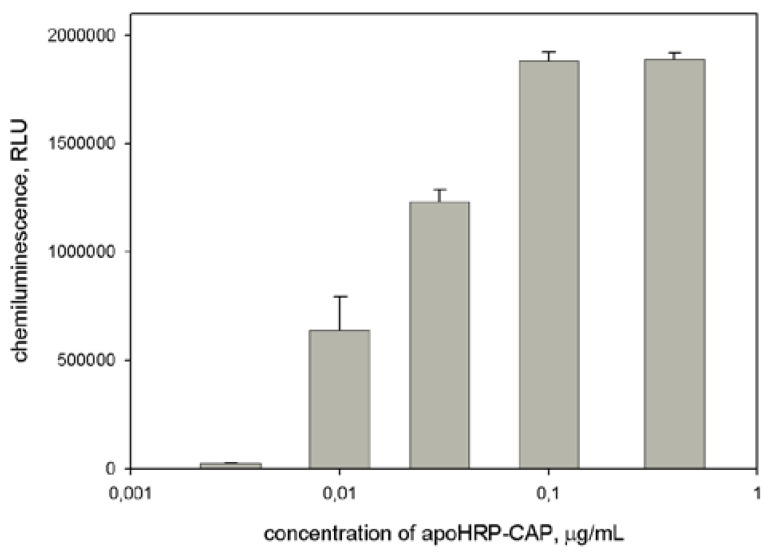
Dependence of light output on concentration of apoHRP-CAP (5:1) at use of biotinylated REP/Stp-HRP as a signaling system. Experimental conditions: concentrations of HBV DNA biotin-REP were 50 pM and 10 pM, respectively; Stp-HRP dilution = 1:500. Chemiluminescence was measured 8 min after the initiation of luminol oxidation. Each measurement was repeated thrice.

**Figure 6:**
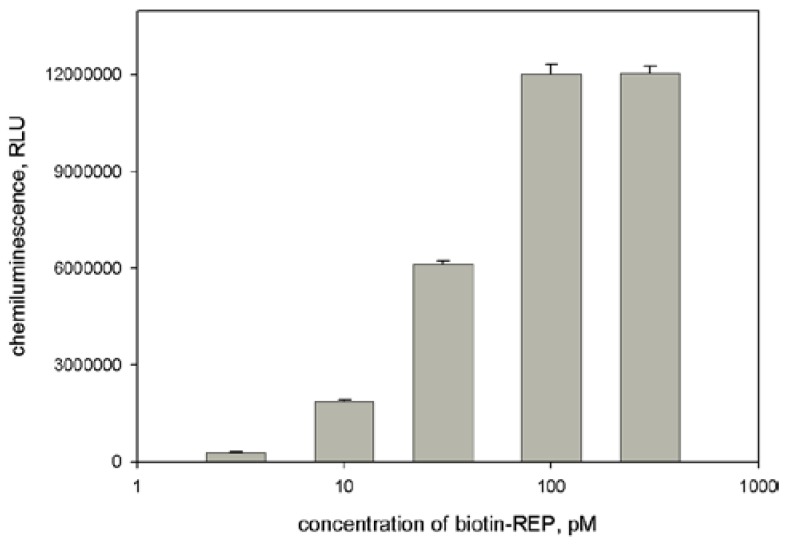
Dependence of light output on concentration of biotinylated reporter. Experimental conditions: concentrations of HBV DNA and apoHRP-CAP (5:1) were 50 pM and 0.1 μg/mL, respectively; Stp-HRP dilution = 1:500. Chemiluminescence was measured 8 min after the initiation of luminol oxidation. Each measurement was repeated thrice.

The alternative signaling system was based on use of biotinylated REP and HRP-streptavidin conjugate (Figure 1B). The experimental conditions of this assay performance were also optimized. Based on the results presented in Figure 5 and Figure 6 we concluded that the favorable conditions for the assay with the second signaling system are the following: 0.1 μg/mL apoHRP-CAP and 0.1 nM biotinylated REP. It should be noted that in the case of biotinylated REP its activity was not decreased by increasing its concentration, whereas for HRP-REP conjugate the bell-shaped effect was observed. In the latter case the decrease of HRP-REP activity likely connects with its aggregation at high concentrations.

Comparison of the favorable conditions for two developed assays showed that at the use of the latter system the concentrations of apoHRP-CAP and REP-containing reagent were in 15 and 100 times lower, respectively, compared with the use of HRP-REP conjugate. At the other hand, it should be noted that at HRP-REP use the analysis time was shorter by one hour. 

### 3.4. Analytical Parameters of HBV DNA Assays

**Figure 7:**
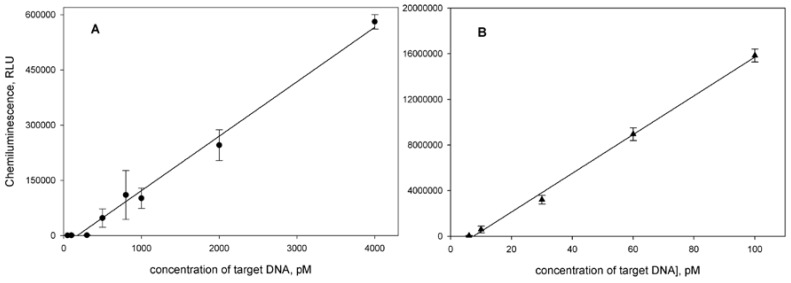
Calibration curves for HBV DNA detection by chemiluminescent sandwich assays using (**A**) HRP-REP conjugate and (**B**) biotinylated REP/Stp-HRP as signaling systems (*n* = 3).

The dependences of light output upon the target concentration allowed us to calculate analytical parameters for the developed assays (Figure 7). For the assay using HRP-REP the detection limit, calculated with 3σ method, and linear range were 50 pM and 300–4000 pM (R^2^ = 0.9914), respectively. The sensitivity of this assay was 158 RLU/pM of target. This assay has not only low sensitivity, but low precision too (CV 10–30%).

At the use of the signaling system consisting of biotinylated REP and HRP-streptavidin conjugate, the detection limit and linear range were 3 pM and 6–100 pM (R^2^ = 0.9971), respectively. The sensitivity of this assay was 1.6 × 10^5^ RLU/pM of target. CV values for determination of HBV DNA concentrations within the working range was lower than 6%. 

Although the reasons of the difference in the activities of the used signaling systems were not studied in detail, we assume that this is connected with steric hindrances arising upon the interaction of HRP-REP conjugate having a large size and the target DNA preliminary bound with apoHRP-CAP conjugate immobilized on microplate. The replacement of HRP (MW 44000) in the reporter molecule with low molecular weight biotin (MW 244.3) significantly reduces the effect of steric hindrances for the interaction of REP and the target and, in turn, improves the analytical parameters of the assay.

## 4. Conclusions

In our work we developed the synthesis of the covalent conjugate of apoHRP and amino-containing oligonucleotides. This synthetic method can likely be used also to produce DNA conjugates with other glycoproteins which have a high content of carbohydrates [46]. Using the obtained conjugate DNA-apoHRP as a capture reagent, a microtiter plate-based assay for detection of HBV DNA was successfully constructed. To detect the target DNA, the signaling system consisted of biotinylated reporter oligonucleotide and HRP-streptavidin conjugate was preferable than HRP-REP conjugate. In order to increase the sensitivity of the proposed assay we applied an enhanced chemiluminescence reaction, where 3-(10′-phenothiazinyl)propane-1-sulfonate/*N*-morpholinopyridine pair was used as enhancer. The obtained results demonstrated that the assay developed with use of microtiter plates has high sensitivity and precision. In further work the DNA assay will be improved using a combination of the proposed assay with isothermal DNA amplification methods.

## Supplementary Materials

The following are available online at http://www.mdpi.com/1424-8220/18/4/1289/s1, Figure S1: UV-vis spectrum of horseradish apoperoxidase.
