# Supplementary material for: Microplate Chemiluminescent Assay for DNA Detection Using Apoperoxidase-Oligonucleotide as Capture Conjugate and HRP-Streptavidin Signaling System
Source: Sensors (Basel). 2018 Apr 23;18(4):1289. doi: 10.3390/s18041289 (PMC5948693; doi:10.3390/s18041289)
Supplement: Supplementary file 1 [file sensors-18-01289-s001.pdf]

Microplate chemiluminescent assays for HBV DNA detection using apoperoxidase-oligonucleotide as capture conjugate and different HRP-containing signaling systems

Ivan Yu. Sakharov

Department of Chemistry, Lomonosov Moscow State University, Leninskie gory, Moscow 119991, Russia, sakharovivan@gmail.com

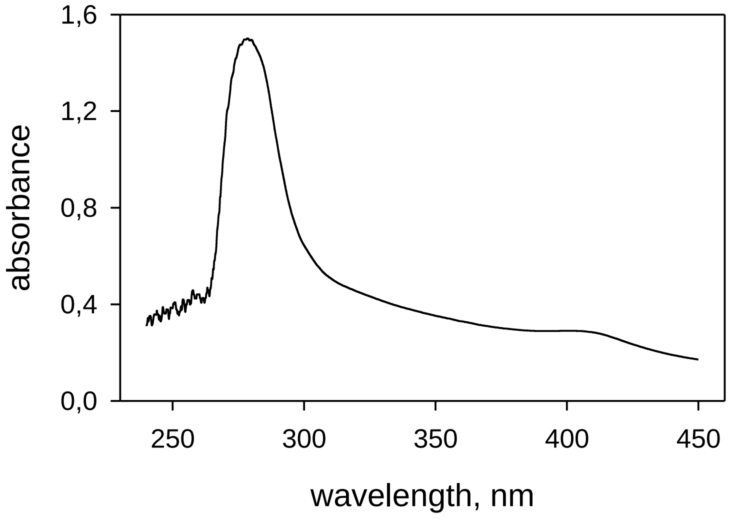

Figure S1. UV-vis spectrum of horseradish apoperoxidase.
